# Abstract not submitted for online publication

**DOI:** 10.1186/1756-8935-6-S1-O5

**Published:** 2013-03-18

## 

Abstract not submitted for online publication

