# Erratum

**DOI:** 10.1186/1756-8935-6-S1-P135

**Published:** 2013-05-30

## 

In volume 6 Supplement 1, Epigenetics and Chromatin: Interactions and processes, abstract O5 was published erroneously. The published record for *Epigenetics & Chromatin*, 2013 **6**(Suppl 1):O5 should read: 'Abstract not submitted for online publication'.
